# Dose- and Time-Dependent Cytotoxicity of Carteolol in Corneal Endothelial Cells and the Underlying Mechanisms

**DOI:** 10.3389/fphar.2020.00202

**Published:** 2020-03-06

**Authors:** Wen Su, Jun Zhao, Ting-Jun Fan

**Affiliations:** Laboratory for Corneal Tissue Engineering, College of Marine Life Sciences, Ocean University of China, Qingdao, China

**Keywords:** carteolol, cytotoxicity, necroptosis, apoptosis, human corneal endothelial cells, feline corneal endothelial cells

## Abstract

Carteolol is a non-selective β-adrenoceptor antagonist used for the treatment of glaucoma, and its abuse might be cytotoxic to the cornea. However, its cytotoxicity and underlying mechanisms need to be elucidated. Herein, we used an *in vivo* model of feline corneas and an *in vitro* model of human corneal endothelial cells (HCECs), respectively. *In vivo* results displayed that 2% carteolol (clinical dosage) could induce monolayer density decline and breaking away of feline corneal endothelial (FCE) cells. An *in vitro* model of HCECs that were treated dose-dependently (0.015625–2%) with carteolol for 2–28 h, resulted in morphological abnormalities, declining in cell viability and elevating plasma membrane (PM) permeability in a dose- and time- dependent manner. High-dose (0.5–2%) carteolol treatment induced necrotic characteristics with uneven distribution of chromatin, marginalization and dispersed DNA degradation, inactivated caspase-2/-8, and increased RIPK1, RIPK3, MLKL, and pMLKL expression. The results suggested that high-dose carteolol could induce necroptosis via the RIPK/MLKL pathway. While low-dose (0.015625–0.25%) carteolol induced apoptotic characteristics with chromatin condensation, typical intranucleosomal DNA laddering patterns, G_1_ cell-cycle arrest, phosphatidylserine (PS) externalization, and apoptotic body formation in HCECs. Meanwhile, 0.25% carteolol treatment resulted in activated caspase-2, -3, -8, and -9, downregulation of Bcl-2 and Bcl-xL, upregulation of Bax and Bad, ΔΨm disruption, and release of cytoplasmic cytochrome c (Cyt.c) and AIF into the cytoplasm. These observations suggested that low-dose carteolol could induce apoptosis via a caspase activated and mitochondrial-dependent pathway. These results suggested that carteolol should be used carefully, as low as 0.015625% cartelol caused apoptotic cell death in HCECs *in vitro*.

## Introduction

Primary-open glaucoma is a chronic optic neuropathy that causes an increase in IOP. If left untreated, primary-open glaucoma can lead to neuropathy and impaired vision (Bartelt-Hofer et al., 2019). Today, the incidence of glaucoma in adults is estimated to be approximately 2–4% in individuals over the age of 40, and its incidence increases with age, according to the Chinese Glaucoma Study Consortium (CGSC; Zhang et al., 2019). Evidence-based mechanisms that can be adopted to prevent and treat glaucoma aim to decrease IOP (Soares and Razeghinejad, 2018).

Carteolol is an agent that can inhibit active secretion or ultrafiltration of aqueous humor from the non-pigmented ciliary epithelium, and in doing so can decrease IOP in the cornea. Thus, carteolol is often used as a non-selective β-adrenoceptor antagonist in the setting of anti-glaucoma treatment in the eye clinic (Nakamoto and Yasuda, 2010). Since glaucoma is a chronic optic neuropathy that requires lifelong daily treatment, several clinical studies have indicated that carteolol abuse can cause uncontrollable side-effects on the cornea, including blurred vision and hypotension in long-acting solution, at least in some patients (Wu et al., 2007; Hiraoka et al., 2011). Moreover, recent studies have found that some same types of beta adrenergic antagonists are serious cytotoxic mediators (Loma et al., 2018). Examples include brimonidine, which can cause hypertensive acute granulomatous anterior uveitis (Clemente-Tomás et al., 2018). Thus, it is necessary to study the cytotoxic effects of such anti-glaucoma drugs.

The cytotoxicity and underlying cellular mechanisms of carteolol on human corneal epithelial cells have been confirmed in prior studies published by our group (Shan and Fan, 2016). However, human corneal epithelium is not the most effective enrichment region of carteolol in the cornea. Meanwhile, corneal epithelial cells might exhibit an ability to self-renew, differentiate, proliferate, and migrate to the central cornea from limbal stem cells due to the fact that the cells are damaged (You J. et al., 2018; You L. et al., 2018). The HCE is composed of a monolayer of hexagonal cells that is located on the innermost layer of the cornea and directly contacted by aqueous humor that is infiltrated by carteolol, which is essential in the maintenance of corneal transparency and pump functions (Li et al., 2019). Furthermore, HCECs lack the potential to proliferate *in vivo* (Bourne, 2003; Joyce, 2012; Meekins et al., 2016). Thus, corneal endothelial cells can yet be regarded as a more relevant and appropriate model to evaluate the cytotoxicity of carteolol eye solutions as compared with corneal epithelial cells. Recently, a non-transfected HCE cell line has been successfully established in our laboratory, which was shown to maintain normal inherent properties and exhibited a normal human karyotype and functional protein expression (Fan et al., 2011). Thus, the non-transfected HCE cell line provides an effective *in vitro* model to explore the toxicological study of carteolol.

In this study, using an *in vivo* model of the feline cornea, and an *in vitro* model of HCECs (Fan et al., 2011), we systematically evaluated the cytotoxicity of carteolol eye solutions in an attempt to provide novel insights into the cytotoxic mechanisms of anti-glaucoma drugs on the cornea. The results of this study should be of great importance in establishing medicinal therapy with carteolol in the setting of the eye clinic.

## Materials and Methods

### Materials

Carteolol powder (C_16_H_25_ClN_2_O_3_; MW: 328.83 Daltons; CAS Registration No: 51781-21-6; Cat No:BP567, Sigma–Aldrich, St. Louis, MO, United States) with a purity greater than 99.0%, was dissolved and filtered in serum-free DMEM/F12 medium (Cat No: R10-092-CV, Corning, NY, United States), and double-diluted with DMEM/F12 medium that included 20% fetal bovine serum (FBS) (Cat No: 10100147, Gibco, NY, United States), at a final concentration of 0.00390625–2% in DMEM/F12 medium, which included 10% FBS before use *in vitro* (Shan and Fan, 2016). The carteolol eye-drop solution was prepared at a clinically relevant therapeutic concentration of 2% by dissolving carteolol power in sterile saline for *in vivo* experiments. Nec-1 was purchased from MedChem Express (Cat No:HY-15760, NJ, United States), and was dissolved in 50 mg/mL DMSO (Cat No:D2650, Sigma–Aldrich, St. Louis, MO, United States) with sterile saline at a stock concentration of 10 mM, and diluted with sterile saline at a concentration of 10 μM before use. HCECs were cultured in DMEM/F12 medium including 10% FBS, at 37°C in a 5% CO_2_ incubator. The HCECs were provided from an established non-transfected HCECs line in our laboratory (Fan et al., 2011).

### Experimental Animals

Twelve healthy male domestic felines without eye or corneal disorders (body weight 2.5 kg, age: 8 months), were purchased from the Experimental Animal Centre of Shandong Province [Jinan, China, Animal permit number: SYXK-(SD)-2013-0001]. They were maintained in an air-conditioned animal room with a temperature of 22 ± 1°C, a relative humidity of 55 ± 5%, ventilation frequency of 18 times/h, and a 12 h light/dark cycle. Each animal was housed in isolated stainless-steel cages and allowed free access to food and water throughout the acclimation period. All animals were acclimated for 1 week prior to initiating experiments, and their use in toxicity studies was approved by the Institutional Animal Care and Use Committee of Shandong Hongli Medical Animal Experimental Research Corporation. All animal protocols complied with the guidelines in the Vision and Ophthalmology (ARVO) statement, for the use of animals in eye and vision research.

### *In vivo* Detection of Feline Corneas

To detect the cytotoxicity of carteolol in endothelial cells, we used an *in vivo* model of feline corneas. The corneas of the right eyes of 12 felines were treated with two drops of 2% carteolol solution (the clinical dosage) twice a day at an interval of 12 h for 7 days in total, while the left eyes were treated with drops of saline solution as blank controls. Corneal ECD was measured using an SP-3000P specular microscope (Topcon, Tokyo, Japan), CCT and IOP were measured using an SW-1000P ultra-sound pachymeter (Souer Electronic Technology, Tianjin, China) and an applanation tonometer (TONO-PEN AVIA, NY, United States), respectively. ECD, CCT, and IOP for each group were assayed on days 0, 7, 14, 21, 28, 35 after treatment with 2% carteolol. The continuous monolayer status of FCE cells was determined by H&E staining of paraffin sections, and intercellular junctions were observed by 1% alizarin red staining. The morphology of FCE cells was detected by a JSM2840 SEM (JEOL, Tokyo, Japan), and the ultrastructure of FCE cells was detected by a H700 TEM (Hitachi, Tokyo, Japan). Apoptosis cells was examined by terminal deoxynucleotidyl transferase-mediated dUTP nick-end labeling (TUNEL) staining with fluorescein isothiocyanate (FITC), using a one-step TUNEL *in situ* cell apoptosis detection kit (Cat No: KGA7032, KeyGen Biotech, Nanjing, China) method and observed under a Nikon E80i fluorescent microscope (Fan and Fan, 2017).

### *In vitro* HCEC Culture and Treatment

The non-transfected HCECs at passage 100 were cultured in DMEM/F12 medium including 10% FBS at 37°C in 25 cm^2^ flasks or plates (Corning, NY, United States) as described previously (Fan et al., 2011). When the cells grew into the logarithmic phase (60–80% confluence), the culture medium was replaced completely with DMEM/F12 medium including 10% FBS. HCECs were supplemented with different concentrations of carteolol that ranged from 0.00390625 to 2% in each flask, respectively. To further confirm the role of high-dose carteolol (0.5–2%) in necroptosis, Nec-1 (10 μM) was treated in conjunction with high-dose carteolol in each flask, respectively. Non-carteolol-treated HCECs were served as blank controls in all of the experiments.

### Morphological Observations

Different concentrations (0.00390625–2%) of carteolol-treated HCECs were seeded into 24-well culture plates at a density of 1 × 10^5^ cells/1 mL/well. HCEC morphology and growth status were monitored by a TS100 inverted microscope (Nikon, Tokyo, Japan) every 2–4 h after treatment with carteolol.

### MTT Assay

The cell viability of HCECs was evaluated by MTT (Cat No:M2128, Sigma–Aldrich, St. Louis, MO, United States) assay (Fan and Fan, 2017). HCECs were seeded into a 96-well plate at a density of 1 × 10^4^ cells/100 μL/well, and were treated with 0.00390625–2% carteolol as described above. Each well was administered 20 μL of 5 mg/mL MTT at an interval of 2–4 h, the plates were then incubated at 37°C in a 5% CO_2_ incubator for 4 h in the dark. Each well was removed and supplemented with 150 μL of DMSO (Cat No: D2650, Sigma–Aldrich, St. Louis, MO, United States), and then measured at a 490 nm absorbance by a Multiskan GO microplate reader (Thermo Scientific, Waltham, MA, United States). The EC_50_ value of carteolol was calculated based on the cell viability of HCECs in the monitoring period of 28 h.

### AO/EB Double-Staining

Plasma membrane permeability of HCECs was detected by AO/EB double-staining (Fan and Fan, 2017). HCECs were seeded into a 24-well plate at a density of 1 × 10^5^ cells/1 mL/well, treated with 0.00390625–2% carteolol and harvested by 0.25% trypsin treatment. Then the cells were resuspended in 0.1 mL of serum-free DMEM/F12 medium and 4 μL of 100 μg/mL AO/EB (1:1) solution (Cat No:A6014/E1510, Sigma–Aldrich, St. Louis, MO, United States), then stained for 1 min, and observed under a Nikon Ti-S fluorescence microscope (Nikon, Tokyo, Japan). The dead cells with red or orange stained-nuclei were designated as having their PM permeabilized, while living cells were designated as having green-stained nuclei. At least 300 cells were counted randomly in each group (*n* = 3). The formula for the PM permeability ratio was:

$$PMpermeability\left(\%\right)=\frac{r\,e\,d\,o\,r\,o\,r\,a\,n\,g\,e\,n\,u\,c\,l\,e\,i\,n\,u\,m\,b\,e\,r}{t\,o\,t\,a\,l\,c\,e\,l\,l\,s\,n\,u\,m\,b\,e\,r}\times 100$$

### DNA Agarose Gel Electrophoresis

DNA diffusion degradation and fragmentation of HCECs was detected by agarose gel electrophoresis (Fan and Fan, 2017). HCECs were cultured in 25 cm^2^ flasks at a density of 1 × 10^6^ cells/5 mL/flask, and harvested as mentioned. After being washed three times by ice-cold PBS and centrifuged (1000 g, 15 min), their total genomic DNA was extracted with a TIANamp Genomic DNA Kit (Cat No:DP304, TianGen Biotechnology, Beijing, China). All DNA samples from each group were electrophoresed in a 1% agarose gel (200 mA, 260 min), the gel was stained with 0.5 μg/mL EB solution for 10 min, and observed with an UVP EC3 imaging system (UVP, Upland, CA, United States).

### Transmission Electron Microscopy Observations

The ultrastructure of HCECs was characterized by TEM (Fan and Fan, 2017). The cells were cultured in 25 cm^2^ flasks at a density of 1 × 10^6^ cells/5 mL/flask, treated with 0.25% carteolol, and harvested at 4, 8, and 12 h, respectively. After fixing in 4% glutaraldehyde and 1% osmium tetroxide, the HCECs were dehydrated, then embedded in epoxy resin, and detected by an H700 TEM (Hitachi, Tokyo, Japan).

### Flow Cytometry Analysis

The cell cycle status, PS externalization in the PM, and ΔΨm of HCECs were all detected by FCM (Fan and Fan, 2017). HCECs in six-well plates at a density of 1 × 10^5^ cells/2 mL/well were treated with 0.25% carteolol and harvested at 4, 8, and 12 h, and fixed in 75% alcohol overnight at 4^*o*^C. The cells were stained in 500 μL PI (Cat No: 11858777001, Roche, Mannheim, Germany) for cell cycle assay, supplemented with 5 μL FITC-labeled Annexin V and 5 μL PI for the PS externalization assay, and 10 μg/mL JC-1 reagent (Cat No: T4069, Sigma–Aldrich, St. Louis, MO, United States) for the ΔΨm assay, respectively. The stained HCECs were detected by an ACEA NovoCyte flow cytometer (ACEA, Hangzhou, China).

### ELISA Analysis

Caspase activation in HCECs was measured by ELISA (Fan and Fan, 2017). To detect the expression of caspase-2 and caspase-8 in pro-necroptotic processes, HCECs were cultured and then treated with 2, 1, and 0.5% carteolol in six-well plates at a density of 1 × 10^5^ cells/2 mL/well for 0, 1, 2, 3, and 4 h. To detect the expression of caspase-2, -3, -8, and -9 in a pro-apoptotic process, HCECs were treated with 0.25% carteolol in six-well plates and harvested at 0, 2, 4, 8, 10, 12, 14, and 16 h post-treatment. Whole-cell protein extracts were prepared with 500 μL RIPA lysis buffer (Cat No:CW2333S, CW biotech, Beijing, China) containing 5 μL PMSF (Cat No:P0100, Solarbio, Beijing, China), which was coated into a high binding 96-well microtiter plate overnight at 4°C. The whole-cell proteins were blocked with 5% non-fat milk (Cat No:232100, BD Bioscience, Franklin Lakes, NJ, United States) at 37°C for 2 h, and incubated with 100 μL rabbit anti-human caspase-2, -3, -8, and -9 (active form) monoclonal antibodies (1:500; Cat No:10436-1-AP, 19677-1-AP, 13423-1-AP, 10380-1-AP, Proteintech, Chicago, IL, United States) at 37°C for 2 h, respectively, and then incubated with 100 μL horseradish peroxidase (HRP)-conjugated goat anti-rabbit secondary antibody (1:2500; Cat No:SA00001-2, Proteintech, Chicago, IL, United States) at 37°C for 1 h, respectively. After each treatment, the samples were washed three washes in PBST, and a colorimetric reaction was induced by 1% TMB (Cat No: PR1200, Solarbio, Beijing, China) at room temperature in the dark for 25 min and terminated with 50 μL of 2 M sulfuric acid solution, following which, each well was measured at an absorbance of 490 nm by a Multiskan GO microplate reader (Thermo Scientific, Waltham, MA, United States) for the quantification of caspase activation.

### Immunochemistry Analysis

The necroptotic proteins RIPK3 and MLKL in HCECs were detected by immunochemistry. HCECs were seeded into 24-well plates at a density of 1 × 10^5^ cells/1 mL/well, and cultured at 37°C in a 5% CO_2_ incubator. When the cells grew to the logarithmic phase, they were fixed in 4% paraformaldehyde for 30 min, and then permeabilized in 500 μL of 0.5% Triton X-100 (Cat No:P1080, Solarbio, Beijing, China) (prepared by PBS) at room temperature for 15 min. Then the cells were blocked in 500 μL of 2% bovine serum albumin (BSA) (Cat No: SW3015, Sloarbio, Beijing, China) (prepared by PBS) at 37°C for 1 h. Samples were then incubated with primary antibody RIPK3 (1:1000, ab56164, Abcam, Cambridge, United Kingdom) and MLKL (1:1000, ab183770, Abcam, Cambridge, United Kingdom) overnight at 4°C, following which, they were incubated with corresponding secondary antibodies for 1 h at room temperature. After each treatment, the samples were washed three washes in PBST. The cells were stained with DAPI (Cat No:C0060, Solarbio, Beijing, China) for 10 min, and then imaged with an E80i positive fluorescence microscope (Nikon, Tokyo, Japan). The immunocytochemistry intensity of each band was analyzed with ImageJ software (NIH, NY, United States).

### Western Immunoblotting Analysis

The expression levels of necroptotic and apoptotic proteins of RIPK1, RIPK3, MLKL, pMLKL, and the Bcl-2 family of proteins, and the cytoplasmic proteins in HCECs, were all quantified by Western immunoblotting (Fan and Fan, 2017). HCECs were treated with 2, 1, and 0.5% carteolol in 25 cm^2^ flasks at a density of 1 × 10^6^ cells/5 mL/flask and harvested at 4 and 8 h post-treatment. The cells were then treated with 0.25% carteolol and harvested at 4, 8, and 12 h. Whole-protein extraction was prepared as described above for ELISA. The phosphorylated protein was prepared using RIPA lysis buffer (Cat No:PC101, EpiZyme, Shanghai, China) and a phosphatase inhibitor (Cat No:78440, Thermo, Waltham, MA, United States), and a mitochondrial/cytoplasmic protein extraction kit (Cat No:P0027, Beyotime, Shanghai, China) was used to extract cytoplasmic proteins. The protein extract in each group was electrophoresed by 12% sodium dodecyl sulfate polyacrylamide gel electrophoresis (SDS-PAGE), and then incubated with 5% skim milk in PVDF membranes (Millipore, Billerica, MA, United States) at room temperature. Next, we incubated the membranes overnight at 4°C with primary antibody to β-actin (1:2500, Cat No: ab8226, Abcam, Cambridge, United Kingdom) as an internal reference control, RIPK1 (1:1000, Cat No:ab106393, Abcam, Cambridge, United Kingdom), RIPK3 (1:1000, Cat No:ab56164, Abcam, Cambridge, United Kingdom), MLKL (1:1000, Cat No:ab183770, Abcam, Cambridge, United Kingdom), pMLKL (1:1000, Cat No: ab187091, Abcam, Cambridge, United Kingdom), Bcl-2 (1:1000, Cat No:12789-1-AP, Proteintech, Chicago, IL, United States), Bcl-xL (1:1000, Cat No: 26967-1-AP, Proteintech, Chicago, IL, United States), Bad (1:1000, Cat No:10435-1-AP, Proteintech, Chicago, IL, United States), Bax (1:1000, Cat No:10783-1-AP, Proteintech, Chicago, IL, United States), Cyt.c (1:1000, Cat No:10993-1-AP, Proteintech, Chicago, IL, United States), and AIF (1:1000, Cat No: 17984-1-AP, Proteintech, Chicago, IL, United States), respectively. Subsequently, they were incubated with corresponding secondary antibodies for 2 h at room temperature. After each treatment, the samples were washed three times in TBST. Finally, the protein bands were combined with an ECL Western Immunoblotting Kit (Cat No:CW0049S, CWbiotech, Beijing, China), and observed under the Tannon Imaging System (Beijing, China). The optical density of each band was analyzed with ImageJ software (NIH, NY, United States), and used β-actin as an internal reference control (*n* = 3).

### Statistical Analysis

All statistical analyses were conducted for results from three independent experiments using the SPSS 20.0 statistical software (SPSS Inc., Chicago, IL, United States). All measurement data were presented as means ± SD (*n* = 3). One-way analysis of variance (ANOVA) with *post hoc* Tukey’s test was chosen to compare data between multiple groups and paired Student’s *t*-test was used to compare data between two groups. Differences with controls were considered statistically significant at an alpha value of *P* < 0.05, and extremely significant at *P* < 0.01.

## Results

### Clinical Carteolol Induced Cytotoxicity of FCE Cells *in vivo*

**FIGURE 1:**
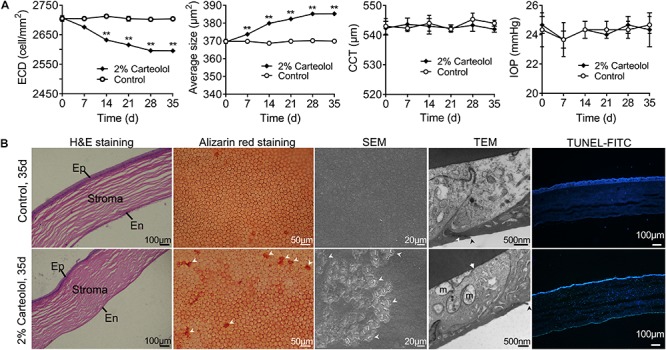
The effects of 2% carteolol for seven days on feline corneas *in vivo*. **(A)** Detection of endothelial cell density (ECD), average size, central corneal thickness (CCT), and intraocular pressure (IOP) after being treated with 2% carteolol. The ECD of 2% carteolol treated eyes decreased, and the average size increased as compared with control eyes (*P* < 0.01). The results showed that CCT and IOP had no obvious differences from those of control eyes (*P* > 0.05). The time-dependent recovery of ECD, average size, CCT and IOP of carteolol eyes, and control eyes are shown, respectively (*n* = 3). **P < 0.01 vs control. **(B)** H&E staining of paraffin-embedded sections showed that the feline cell endothelium was shed after being treated with 2% carteolol, Ep: epithelium, En: endothelium. Alizarin red staining revealed that 2% carteolol-treated feline endothelial cells were larger than those of control eyes. The cell structure was disordered and the microvilli were decreased on the cell surface as compared with normal eyes by SEM observation. Moreover, the intercellular tight junctions of corneal endothelial cells and the mitochondria were swollen as seen by TEM observations, m: swollen mitochondrion, arrow: impaired cell junction; Apoptosis cells was examined by terminal deoxynucleotidyl transferase-mediated dUTP nick-end labeling (TUNEL) staining with fluorescein isothiocyanate (FITC) fluorescent dye, with nuclei counterstained with 4′,6-diamidino-2-phenylindole (DAPI). Images represent *n* = 3.

The toxic effect of carteolol on feline corneal endothelial (FCE) cells was investigated using an *in vivo* model of feline corneas following exposure to 2% carteolol for 7 days. The results showed that the ECD of carteolol-treated eyes decreased, and the average cell size had increased as compared with control eyes (*P* < 0.01; Figure 1A), while the CCT and IOP were unaltered as compared with control eyes. H&E staining showed that FCE cells were shed on treatment with 2% carteolol. Alizarin red staining revealed that carteolol-treated FCE cells were slightly larger than control eyes. The effect of carteolol on the ultrastructure of FCE cells was evaluated using electron microscopy. The cell structure was shown to be disordered and microvilli were decreased on the cell surface as compared normal eyes by SEM. Moreover, the intercellular tight junction of corneal endothelial cells was destroyed and the mitochondria were swollen as studied by TEM. Furthermore, most of the corneal endothelial cells of carteolol treated feline corneal tissue were positive staining following TUNEL examination, which indicated that their nuclear DNA were fragmented (Figure 1B). Observations indicated that twice a day 2% carteolol for 7 days was cytotoxic to corneal endothelium and had apoptosis-inducing effect on FCE cells *in vivo*.

### Cytotoxicity Assessment of HCECs *in vitro*

**FIGURE 2:**
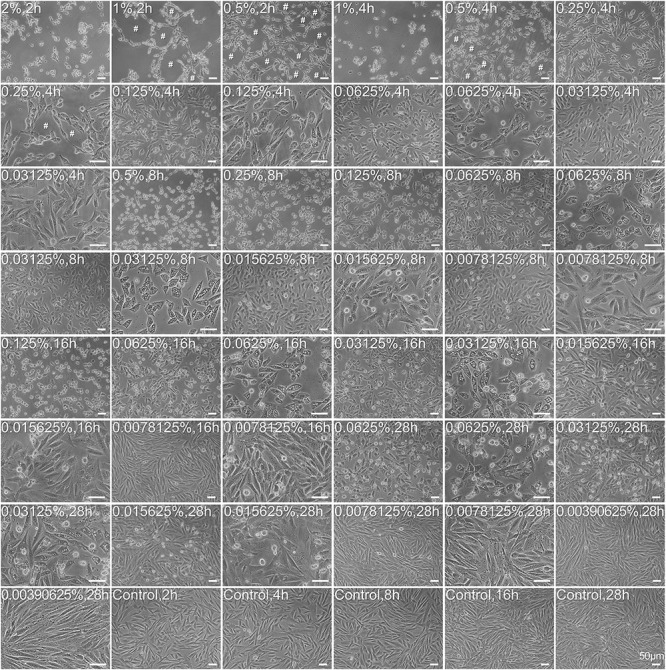
Morphological abnormality and growth status of carteolol-treated HCECs. The concentration and exposure time of carteolol were indicated on the top-left of each image. Images represent *n* = 3. # denotes the cytopathic effect. Scale bar is 50 μm.

The morphology of carteolol-treated HCECs was observed by light microscopy. The results showed that carteolol-exposed HCECs at doses that ranged from 0.0015625 to 2% showed cell shrinkage and vacuolization. HCECs that were treated with 0.5–2% carteolol were exfoliated at a short time for 2, 4, and 8 h, and at 0.015625–0.25% carteolol cells were found to be vacuolized beginning at 4 h. In addition, along with the extension of time, the degree of vacuolation increased, while those treated with 0.0078125 and 0.00390625% carteolol showed no obvious difference as compared with blank controls (Figure 2 and Supplementary Figure S1). These results implied that above 0.0015625%, carteolol displayed dose- and time-dependent cytotoxicity against HCECs *in vitro*.

### Carteolol Induced Cell Viability Decrease and PM Abnormality of HCECs

**FIGURE 3:**
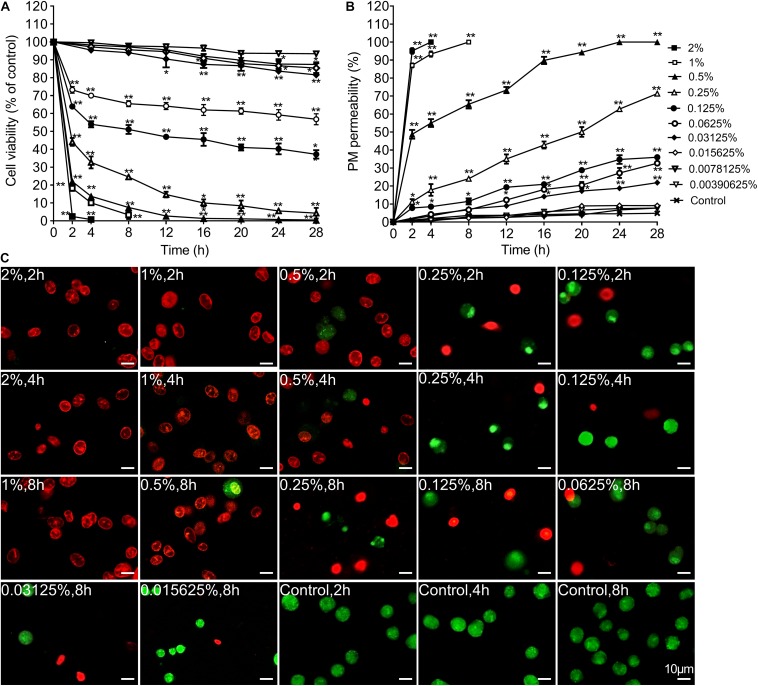
The time and concentration dependent effect of carteolol on the cell viability and plasma membrane permeability. **(A)** MTT assay. The cell viability of each group treated with different concentrations and exposure times of carteolol was expressed as a percentage (mean ± SD) as compared to its corresponding control (*n* = 3). *P < 0.05, and **P < 0.01 vs control. **(B)** AO/EB double-staining assay. HCECs treated with or without the indicated concentrations and exposure times of carteolol. The necroptosis and apoptotic ratio of each group was expressed as a percentage (mean ± SD) of the total number of cells based on elevated permeability of the plasma membrane (*n* = 3). *P < 0.05, and **P < 0.01 vs control. **(C)** AO/EB double-staining images of carteolol-treated HCECs. The dead cells with red or orange stained-nuclei were designated as having their PM permeabilized, while living cells were designated as having green-stained nuclei. Scale bar is 10 μm.

To validate the cytotoxicity of carteolol, cell viability and PM permeability of carteolol-treated HCECs was detected by the MTT assay and AO/EB double-staining. The results found that cell viability decreased in HCECs after treatment with the concentrations above 0.0015625% (*P* < 0.01 or 0.05; Figure 3A) in a dose- and time-dependent manner (EC_50_-4 h: 0.5707 mg/mL). Among this, cell viability decreased sharply by 2, 4, and 8 h in the 0.5–2% carteolol-treated group, while cell viability decreased slowly in 0.015625–0.25% carteolol-treated groups, and showed no changes in the 0.00390625–0.0078125% carteolol-treated group as compared with blank controls. AO/EB double results found that carteolol increased PM permeability in HCECs, and did so dose- and time-dependently at concentrations above 0.015625% carteolol as compared the control (Figure 3B). Furthermore, 0.5–2% carteolol treatment induced acute increases in PM permeability with necrotic features, such as an uneven distribution of chromatin and marginalization of non-condensed chromatin (Figure 3C), while low-dose (0.015625–0.25%) carteolol treatment induced apoptotic features of visible chromatin condensation (Figure 3C and Supplementary Figure S2).

### Carteolol Induced Changes in DNA Integrity of Treated HCECs

**FIGURE 4:**
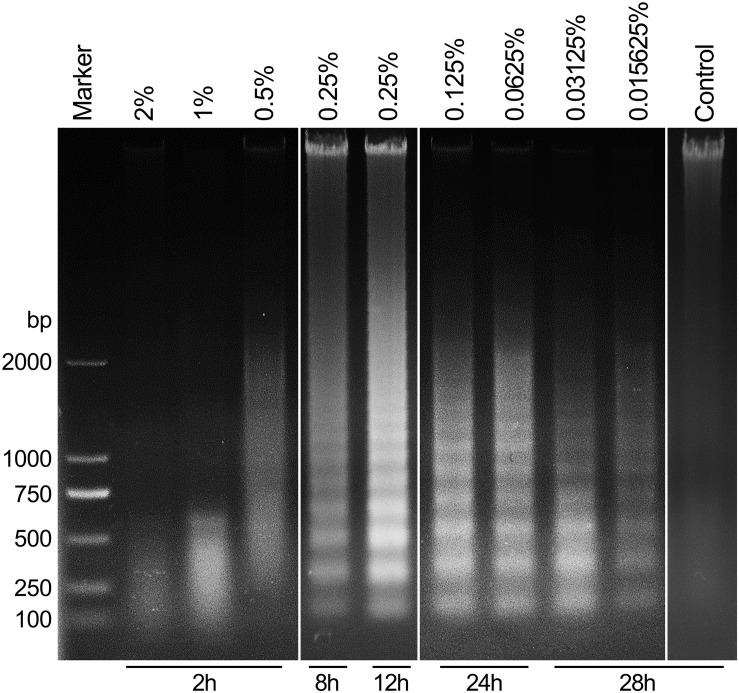
Carteolol-induced DNA diffused degradation and DNA fragmentation in HCECs. DNA that was isolated from HCECs treated with or without the indicated concentration and exposure time of carteolol treatment was electrophoresed in a 1% agarose gel, and the emerged DNA ladders are shown.

To further explore the differential mechanisms of carteolol in a dose- and time-dependent approach, DNA fragmentation of carteolol-treated HCECs was studied by DNA agarose gel electrophoresis. The results showed that degradation of DNA was seen when extracted from high-dose carteolol treated HCECs, and showed dispersed DNA degradation, while low-dose carteolol treated HCECs showed typical intranucleosomal DNA laddering patterns (Figure 4). Thus, when combined with the augmented PM permeability and DNA fragmentation experiments, these data likely confirmed that high-dose carteolol treatment could induce necroptosis, while low-dose carteolol treatment could induce apoptosis.

### Study of Necroptosis-Associated Molecules in High-Dose Carteolol-Treated HCECs

**FIGURE 5:**
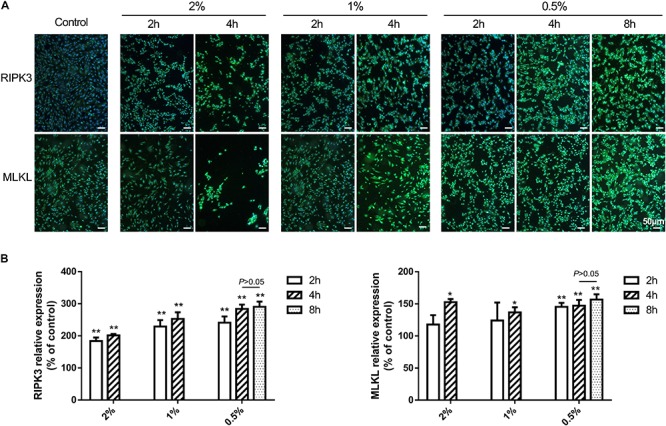
The effect of 0.5–2% cartelol on the pro-necrosome protein expression in HCECs. **(A)** 0.5–2% carteolol upregulated the expression of RIPK3 and MLKL. Scale bar is 50 μm. **(B)** The fluorescence intensity expression of RIPK3 and MLKL was compared to that of the control (*n* = 3). *P < 0.05, and **P < 0.01 vs control.

**FIGURE 6:**
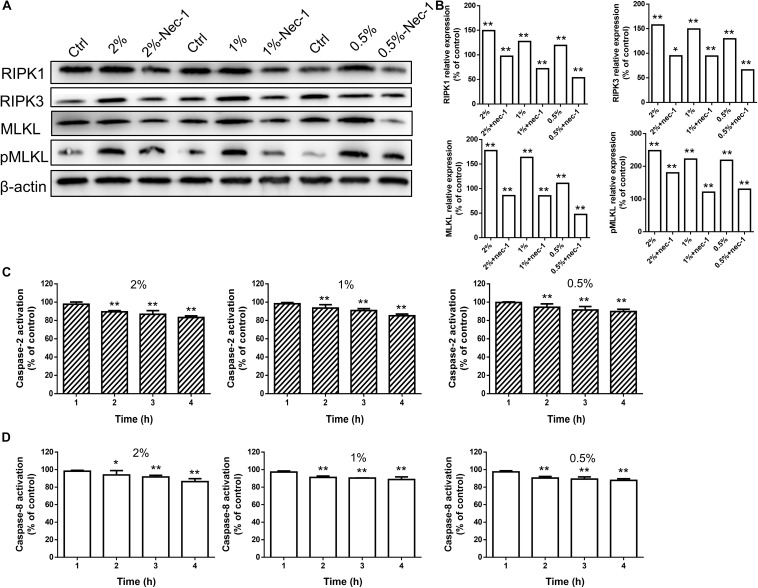
High-dose carteolol induced necroptotic protein expression in HCECs. **(A)** Western immunoblotting images of RIPK1, RIPK3, MLKL and pMLKL proteins in 0.5–2% carteolol-treated HCECs. **(B)** The protein expression of RIPK1, RIPK3, MLKL and pMLKL were upregulated (*P* < 0.01), and the relative amount of each protein was expressed as a percentage (mean ± SD) of protein band density as compared that of β-actin at the same time-point (*n* = 3), while Nec-1 management manifested a significant inhibitory effect on the increased production of these proteins (*P* < 0.05 at the protein expression of RIPK3 with 2% cartelol-treated group, *P* < 0.01 at the other group). *P < 0.05, and **P < 0.01 vs control. **(C)** The activation of caspase-2 was measured by ELISA using monoclonal antibodies targeted against their active forms (*P* < 0.01). The 490 nm absorbance of each group was expressed as mean ± SD (*n* = 3). **P < 0.01 versus the blank control. **(D)** The caspase-8 was measured by ELISA using monoclonal antibodies targeted against their active forms (*P* < 0.05 at the caspase-8 activation with 2% cartelol-treated 2 h group, *P* < 0.01 at the other group). The 490 nm absorbance of each group was expressed as mean ± SD (*n* = 3). *P < 0.05, and **P < 0.01 versus the blank control.

The fluorescence intensity of necroptotic proteins RIPK3 and MLKL was examined by immunocytochemistry. Compared with blank controls, the expression of RIPK3 and MLKL was increased in a time-dependent manner at concentrations greater than 0.5% (*P* < 0.01 or 0.05), while carteolol demonstrated no significant difference at 4 and 8 h in 0.5% carteolol-treated HCECs (*P* > 0.05, Figures 5A,B and Supplementary Figure S4). Therefore, 4 h treated 0.5–2% carteolol exposed HCECs were selected to detect RIPK/MLKL protein expression. Western immunoblotting analysis showed that the protein expression levels of RIPK1, RIPK3, MLKL, and pMLKL were all increased (*P* < 0.01 or 0.05; Figures 6A,B), meanwhile the activity of caspase-2 and caspase-8 were downregulated after 0.5–2% carteolol treatment for 1–4 h as compared with controls (*P* < 0.01 or 0.05; Figures 6C,D). Our research has preliminary implied that carteolol could induce necroptosis in HCECs at concentrations from 0.5 to 2%.

### Necrostain-1 Prevented High-Dose Carteolol-Treated Necroptosis-Associated Molecular Activity in HCECs

Necrostatin-1, which is a small-molecule inhibitor of necroptosis, has been characterized by its selective inhibition of RIPK1 (Degterev et al., 2008). Levels of proteins involved in the RIPK/MLKL signaling pathway were also determined by Western immunoblotting to further confirm the role of high-dose carteolol in necroptosis. The expression levels of RIPK1, RIPK3, MLKL, and pMLKL were significantly increased when compared with the control (*P* < 0.01 or 0.05; Figures 6A,B), while Nec-1 management manifested a significant inhibition of the increased production of these proteins (*P* < 0.01; Figures 6A,B). Data suggest that high-dose carteolol (0.5–2%) induces necroptosis in HCECs.

### Study of Apoptosis-Associated Molecules in Low-Dose Carteolol-Treated HCECs

Combined with the above experimental results, low-dose (0.0015625–0.25%) carteolol treatment induced apoptosis in HCECs, wherein it was found that treatment with 0.25% carteolol was considered the maximum concentration that could induce apoptosis in HCECs. Thus, 0.25% carteolol treatment was appropriate to study the effects of low-dose carteolol treatment and the apoptotic mechanism in HCECs.

**FIGURE 7:**
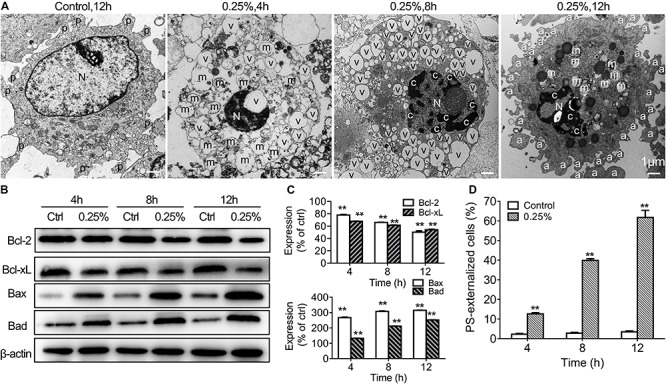
Low-dose carteolol induced ultrastructural abnormality, Bcl-2 family proteins, and PS externalization in HCECs. **(A)** TEM images are shown. HCECs were treated with or without 0.25% carteolol for the indicated times, and their ultrastructure was observed under a TEM. In the images the following are shown: a, apoptotic body; c, chromatin condensation; m, swollen mitochondrion; N, nucleus; v, vacuole; and mv, microvillus. Scale bar is 1 μm. Quantitative alterations in mitochondrion-associated pro-apoptotic regulators are shown. Cultured HCECs were treated with 0.25% carteolol for the indicated times. **(B)** Western immunoblotting images of Bcl-2 family proteins in carteolol-treated HCECs. **(C)** Western immunoblotting analysis of Bcl-2 family proteins in HCECs. Densitometric analysis of protein bands using β-actin as an internal control. The relative amount of each protein was expressed as a percentage (mean ± SD) of respective protein band densities as compared to its corresponding controls (*n* = 3) (*P* < 0.01). **P < 0.01 vs control. **(D)** FCM assay using Annexin-V/PI staining is shown. HCECs were treated with or without 0.25% carteolol for the indicated times, and the cell number of apoptotic cells, i.e., Annexin-V positive PS-externalized cells, was expressed as a percentage (mean ± SD) of the total number of cells (*n* = 3) (*P* < 0.01). **P < 0.01 vs control.

The cell cycle status of carteolol-treated HCECs by PI staining and analysis by FCM was used to verify the apoptotic molecular mechanism of carteolol. After 0.25% carteolol treatment of HCECs for 4, 8, and 12 h, the number of HCECs in the G_1_ phase of the cell cycle increased with time (*P* < 0.01), whereas those in the S phase decreased with time (*P* < 0.01 or 0.05). By contrast, the control group displayed no changes in either the G_1_ or S phases of the cell cycle at the same time interval (Table 1 and Supplementary Figure S5), which implied that carteolol could create a G_1_ phase arrest in HCECs. TEM showed that apoptosis-like ultrastructural changes were seen, for example, microvillus loss, cytoplasmic vacuolation, mitochondrial swelling, chromatin condensation, and apoptotic body formation, as compared with the controls (Figure 7A). Annexin V/PI staining showed that the number of Annexin V-positive cells (PS-externalized cells) increased with time following exposure to 0.25% carteolol for 4, 8, and 12 h (*P* < 0.01, Figure 7D and Supplementary Figure S6). The results suggested that low-dose carteolol exhibited an apoptotic effect on HCECs.

**TABLE 1:** The cell cycle parameters of carteolol-treated human corneal endothelial cells.

	**Number of cells**		
	**G1 phase**	**S phase**	**G2/M phase**
**Control**			
0 h	36.27 ± 0.15	56.31 ± 0.17	6.90 ± 0.32
4 h	37.20 ± 1.39	46.36 ± 1.36	16.38 ± 0.79
8 h	43.92 ± 1.81	40.38 ± 1.93	15.02 ± 0.40
12 h	44.86 ± 1.80	36.83 ± 3.77	17.72 ± 1.64
**0.25% carteolol**			
4 h	45.34 ± 1.92**	40.70 ± 0.94*	13.66 ± 1.29
8 h	58.01 ± 0.95**	31.75 ± 2.00**	10.13 ± 0.96
12 h	63.60 ± 1.15**	22.72 ± 1.10**	13.53 ± 0.13

Assayed by FCM using PI staining. The HCEC numbers in each cell cycle phase was represented as the percent (mean ± SD) of the total number of cells (*n* = 3); *P < 0.05, and **P < 0.01 vs control.

**FIGURE 8:**
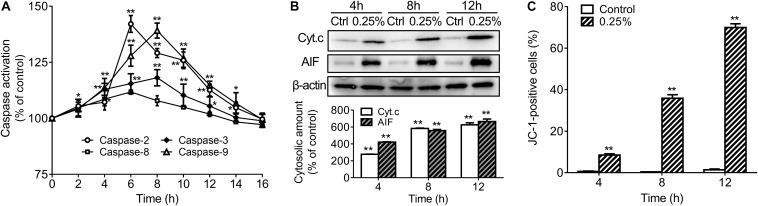
Low-dose carteolol induced caspase activation, quantitative alterations in mitochondrion-associated pro-apoptotic regulators, and ΔΨm disruption in HCECs. **(A)** ELISA detection. HCECs were treated with 0.25% carteolol for the indicated times, and caspase activation was measured by ELISA using the active form of caspase-2/-3/-8/-9 antibodies. The activation ratio of each group was expressed as a percentage (mean ± SD) as compared to its corresponding control based on the absorbance at 490 nm (*n* = 3), *P < 0.05, and **P < 0.01 vs control. **(B)** Western immunoblotting analysis of mitochondria released pro-apoptotic proteins in HCECs. Densitometric analysis of protein bands using β-actin as an internal control. The relative amount of each protein was expressed as a percentage (mean ± SD) of protein band density as compared to its corresponding controls (*n* = 3) (*P* < 0.01). **P < 0.01 vs control. Cyt. c, cytochrome c; AIF, apoptosis inducing factor. **(C)** FCM using JC-1 staining. HCECs were treated with or without 0.25% carteolol for the indicated periods of time, and their ΔΨm disruption was evaluated based on the amount of JC-1 monomer. The number of JC-1 positive cells in each group was expressed as a percentage (mean ± SD) of the total number of cells (*n* = 3) (*P* < 0.01). **P < 0.01 vs control

In order to further explore the apoptotic mechanisms of HCECs, we also further studied activation of caspases-2, -3, -8, -9 in 0.25% carteolol-treated HCECs by ELISA. It was found that caspases-2, -3, -8, and -9 from 0.25% carteolol-treated HCECs were all activated (*P* < 0.01 or 0.05, Figure 8A), wherein it was also found that caspase-2 was first activated to its peak at 4 h (*P* < 0.01, Figure 8A), and caspase-9 was found to have peaked at 6 h (*P* < 0.01, Figure 8A). Notably, the expression of the anti-apoptotic proteins Bcl-2 and Bcl-xL was downregulated (*P* < 0.01, Figures 7B,C), while that of the pro-apoptotic proteins Bax and Bad were increased following treatment of HCECs with 0.25% (*P* < 0.01; Figures 7B,C). The expression of the cytoplasmic levels of the mitochondrial-released pro-apoptotic proteins Cyt.c and AIF were also upregulated following treatment of HCECs with 0.25% (*P* < 0.01, Figure 8B). Low-dose carteolol induced ΔΨm disruption of HCECs, while the number of JC-1 positive cells increased from 0.64 ± 0.16% to 8.49 ± 0.6% (4 h), and 35.87 ± 1.71% (8 h), 69.94 ± 1.81% (12 h) for 4 h, 8 h and 12 h, respectively (Figure 8C and Supplementary Figure S7). Collectively, these data suggested that low-dose carteolol induced HCECs apoptosis, and did so via caspase activation and a mitochondrial-dependent pathway, via caspase activation, dampened expression of the anti-apoptotic protein Bcl-2 and Bcl-xL, enhanced expression of the pro-apoptotic proteins Bax and Bad, and mitochondrial-released pro-apoptotic proteins Cyt.c and AIF.

## Discussion

Carteolol is a major anti-glaucoma therapeutic drug, and it has been reported that prolonged and repeated use of anti-glaucoma medications probably provoke side-effects on HCECs, including low cell survival capacity, which imparts deep concern with regard the clinical safety of this therapeutic agent (Igarashi et al., 1990; Ayaki et al., 2008, 2009; Nagai et al., 2018; Fuwa et al., 2019). Thus, research on the cytotoxic effects and the involved toxicological mechanisms of carteolol on corneal endothelial cells, both *in vivo* and *in vitro*, have crucial importance in providing reference points for the secure administration of carteolol in the eye clinic.

To evaluate the cytotoxicity of carteolol, we first evaluated the cytotoxicity of carteolol using an *in vivo* model. Since cornea endothelial cells from feline species have no proliferation (Landshman et al., 1987), they are considered similar to HCECs, which thus provide a great model to assess the cytotoxicity of carteolol. Our results showed that 2% clinical carteolol induces a time-dependent reduction in corneal ECD, and increases the average size ratio of endothelial cells *in vivo*, which can cause deterioration in visual acuity in ophthalmic disease (Lyssek-Boroń et al., 2019). Furthermore, ultrastructural observations showed that FCE cells had shrunk, and this process was accompanied by mitochondrial swelling and cytoplasmic vacuolation after carteolol treatment (Li W. J. et al., 2016). These results showed that carteolol infiltrate the endothelium, display cytotoxicity against FCE cells *in vivo*, an observation which is of great significance in studying the cytotoxicity and underlying mechanisms of carteolol in terms of its clinical value.

Based on *in vivo* research, we found it necessary to further investigate the cytotoxicity and cellular mechanisms of carteolol in an *in vitro* model of HCECs. Our results showed that carteolol at a concentration above 0.015625% induced morphological abnormality, including cell shrinkage, cytoplasmic vacuolation. This was accompanied by decreased cell viability in a dose- and time-dependent manner, especially in the 0.5–2% carteolol-treated group (the same results in Supplementary Figure S3). Furthermore, the double-fluorescence staining of AO/EB showed that carteolol increased PM permeability of HCECs, and did so in a dose- and time-dependent manner at concentrations above 0.015625% for carteolol therapy. Compared with the low-dose (0.015625–0.25%) carteolol-treated group, the high-dose (0.5–2%) carteolol-treated group induced an acute elevation in PM permeability with an uneven distribution of chromatin, marginalization, and irregular DNA fragmentation, which was typical of necroptosis (Jabłońska-Trypuć et al., 2019). Low-dose carteolol-treated HCECs displayed heightened PM permeability (Leite et al., 1999), DNA ladders (Walker and Sikorska, 1997), condensed chromatin (Moss et al., 2006), and apoptotic body formation (Moss et al., 2006), which were typical apoptosis features. In addition, according to the different features, the cytotoxicity of carteolol and its precise mechanisms were different in the high-dose vs the low-dose carteolol-treated group. Moreover, high-dose carteolol was associated with the induction of cell necroptosis, while low-dose carteolol was associated with the induction of cell apoptosis.

Necroptosis is a form of regulated cell death necrosis, which critically depends on the receptor-interacting RIPK1/3-mediated MLKL containing complex, which leads to phosphorylation and oligomerization of RIPK3, then subsequent activation of RIPK3 mediated MLKL oligomerization and phosphorylation (Sun et al., 2012;Zhao et al., 2012), and finally transfer to the inner leaflet of the PM to execute necroptosis (Wang et al., 2014). Caspase-8 is a key mediator of necroptosis by the RIPK1/RIPK3 necrosome. Under conditions where the activity of caspase-8 is inhibited, RIPK1 is induced, whereupon it binds to RIPK3 and MLKL to form a necrosome, a process that leads to necroptosis (Green et al., 2011; Dillon et al., 2014; Chen et al., 2017). Caspase-2 is also a negative factor of necroptosis (Zamaraev et al., 2018). Our results showed that the protein expression levels of RIPK1, RIPK3, MLKL, and pMLKL were significantly increased as compared with the blank group, and combined with decrease of the activity of caspase-8 and caspase-2, the observations collectively confirmed that high-dose carteolol initiate the necroptosis program.

Necrostatin-1 is a small-molecule inhibitor of necroptosis, which is characterized by its selective inhibitory impact on RIPK1 (Degterev et al., 2005, 2008). Numerous studies have recently discovered the neuroprotective effects of Nec-1, whereas the addition of Nec-1 significantly decreased the related RIPK/MLKL protein levels in necroptosis (Chen et al., 2019). Our results showed that Nec-1 administration significantly dampened the increased expression of RIPK1, RIPK3, MLKL, and pMLKL. Therefore, our results showed that carteolol, at concentrations that ranged from 0.5 to 2% provoked necroptosis in HCECs, by activating the RIPK/MLKL pathway.

Typically, features of apoptosis include PM permeability elevation (Leite et al., 1999), intranucleosomal DNA fragmentation (Walker and Sikorska, 1997), PS externalization (Zwaal and Schroit, 1997), ultrastructural abnormalities (White and Cinti, 2004), and cell cycle retardation (Hengarntner, 2010). Our results showed that 0.25% carteolol-treated HCECs displayed G_1_ cell-cycle phase arrest and externalization of membrane phospholipid PS, and ultrastructural disorganization, chromatin condensation, and the presence of many apoptotic bodies, which were all observed in carteolol-treated HCECs, and which were consistent with typical features of apoptosis.

Apoptosis is conventionally thought to be initiated by two signaling pathways, which include the mitochondrial-mediated endogenous pathway of apoptosis, and the death receptor-mediated exogenous pathway of apoptosis (Wang et al., 2019), both of which can result in the activation of initiator caspases (i.e., caspase-2, -8, -9, and -10), and then the executor caspases (caspase-3, -6, and -7) (Kumar and Vaux, 2002; Fan et al., 2005; Galluzzi et al., 2016). Caspase-2 activation in response to DNA damage provides an important link between such damage and the engagement of the apoptotic pathway (Prasad et al., 2006; Harashima et al., 2014). Caspase-8 mediated the regulation of the TNFR1-mediated extrinsic apoptosis pathway (Tummers and Green, 2017), and Caspase-9 is a key regulator of the mitochondrial-dependent endogenous pathway (Ren et al., 2019). Caspase-9 subsequently activates Caspase-3, which is the most important executor protein in the mitochondrial-mediated apoptosis pathway, and eventually leads to inactivation of PARP (Li Z. Y. et al., 2016). Results showed that 0.25% carteolol stimulates activation of caspase-2, -3, -8, -9, and does so in proper order in HCECs. Our present study demonstrated that caspases were involved in regulating two pathways, namely, the extrinsic and intrinsic pathways of apoptosis that were induced by carteolol.

In combination with caspase activation, our results showed that apoptosis requires the mitochondrial-dependent pathway. It has been demonstrated that the regulation of apoptosis requires mitochondria and the anti-apoptotic Bcl-2 family of proteins (i.e., Bcl-2 and Bcl-xL), pro-apoptotic Bcl-2 family proteins (i.e., Bax and Bad), and other apoptotic trigger proteins isolated from mitochondria. Bcl-2 could bind to mitochondria and prevent the movement of Cyt.c from mitochondria to the cytoplasm (Prenek et al., 2017). Further, Bax could migrate to the outer mitochondrial membrane and enhance mitochondrial permeability, which promotes decreased ΔΨm and Cyt.c release (Rasola and Bernardi, 2007; Kureel et al., 2016). AIF is the key inducing factor of caspase-independent peripheral chromatin and large-scale DNA fragmentation in apoptosis (Cai et al., 1998; Miramar et al., 2001; Wang, 2001; Candé et al., 2002; Zhou et al., 2015). Our results showed that carteolol downregulates the expression of Bcl-2 and Bcl-xL, upregulates the expression of Bax and Bad, and upregulates the mitochondrial release of Cyt.c and AIF in the cytoplasm, respectively. These results, combined with knowledge of the activation of caspases-2, -3, -8, and -9 suggest that low-dose carteolol can induce apoptosis by caspase activated and mitochondrial dependent pathways in HCECs *in vitro*.

## Conclusion

In summary, the cytotoxicity of 2% carteolol was confirmed in models of FCE cells *in vivo*. At concentrations exceeding 0.015625%, carteolol demonstrated a dose- and time-dependent cytotoxic effect on HCECs *in vitro*. High-dose cytotoxicity of carteolol induced necroptosis of HCECs via the RIPK/MLKL pathway. Low-dose cytotoxicity mediated by carteolol on HCECs was achieved by inducing caspase activation and the mitochondrial-dependent pathway.

Collectively these findings provide a novel approach to study toxic effects of external anti-glaucoma drugs for clinical applications. The results of this study suggest that carteolol should be used carefully, as low as 0.015625% cartelol caused apoptotic cell death in HCECs *in vitro*.

## Data Availability Statement

All datasets generated for this study are included in the article/Supplementary Material.

## Ethics Statement

The animal study was reviewed and approved by the Institutional Animal Care and Use Committee of Shandong Hongli Medical Animal Experimental Research Corporation.

## Author Contributions

All listed authors made significant contributions to this manuscript through study design, data analysis and acquisition, or during the process of writing and revisions. WS performed the research, data analysis, and prepared the manuscript. JZ performed the manuscript and revisions and reviewed the manuscript. T-JF was involved in conception and design and data analysis, and approved the final submitted manuscript. All authors commented on previous versions of the manuscript and approved the final manuscript.

## Conflict of Interest

The authors declare that the research was conducted in the absence of any commercial or financial relationships that could be construed as a potential conflict of interest.

## Supplementary Material

FIGURE S1 Morphological abnormality and growth status of carteolol-treated HCECs. The concentration and exposure time of carteolol were indicated on the top-left of each image. Images represent *n* = 3. Scale bar is 50 μm.

FIGURE S2 AO/EB double-staining images of carteolol-treated HCECs. Scale bar is 100 μm.

FIGURE S3 LDH double-staining images of carteolol-treated HCECs.

FIGURE S4 Immunochemistry images of high concentration carteolol-treated the expression of RIPK3 and MLKL protein in HCECs. Scale bar is 50 μm.

FIGURE S5 PI staining by FCM images of cell cycle parameters in 0.25% carteolol-treated HCECs.

FIGURE S6 Annexin-V/PI staining by FCM images of PS-externalized cells.

FIGURE S7 JC-1 staining by FCM images of ΔΨm disruption in 0.25% carteolol-treated HCECs.

## Abbreviations

AIF: apoptosis-inducing factor
AO/EB: acridine orange/ethidium bromide
CCT: central corneal thickness
Cyt.c: cytochrome c
DMEM/F12: Dulbecco’s modified Eagle medium: Ham’s nutrient mixture F-12 medium (1:1)
ECD: endothelial cell density
ELISA: enzyme-linked immunosorbent assay
FCM: flow cytometry
HCE: human corneal endothelium
HCECs: human corneal endothelial cells
H&E: hematoxylin and eosin
IOP: intraocular pressure
JC-1, 5,5’6,6’-tetrachloro-1,1’3: 3’-tetraethyl-imidacarbocyanine
ΔΨ m: mitochondrial transmembrane potential
MLKL: mixed lineage kinase domain like pseudokinase
MTT: methyl thiazolyl tetrazolium
Nec-1: necrostatin-1
PI: propidium iodide
PM: plasma membrane
pMLKL: phosphorylation mixed lineage kinase domain like pseudokinase
PMSF: phenylmethylsulfonyl fluoride
PS: phosphatidylserine
RIPA: radio immunoprecipitation assay
RIPK1: receptor-interacting serine/threonine-protein kinase 1
RIPK3: receptor-interacting serine/threonine-protein kinase 3
SEM: scanning electron microscope
TEM: transmission electron microscope
TUNEL: terminal-deoxynucleoitidyl transferase-mediated nick-end labeling.
