# Supplementary figures and images for: Dose- and Time-Dependent Cytotoxicity of Carteolol in Corneal Endothelial Cells and the Underlying Mechanisms
Source: Front Pharmacol. 2020 Mar 6;11:202. doi: 10.3389/fphar.2020.00202 (PMC7068677; doi:10.3389/fphar.2020.00202)

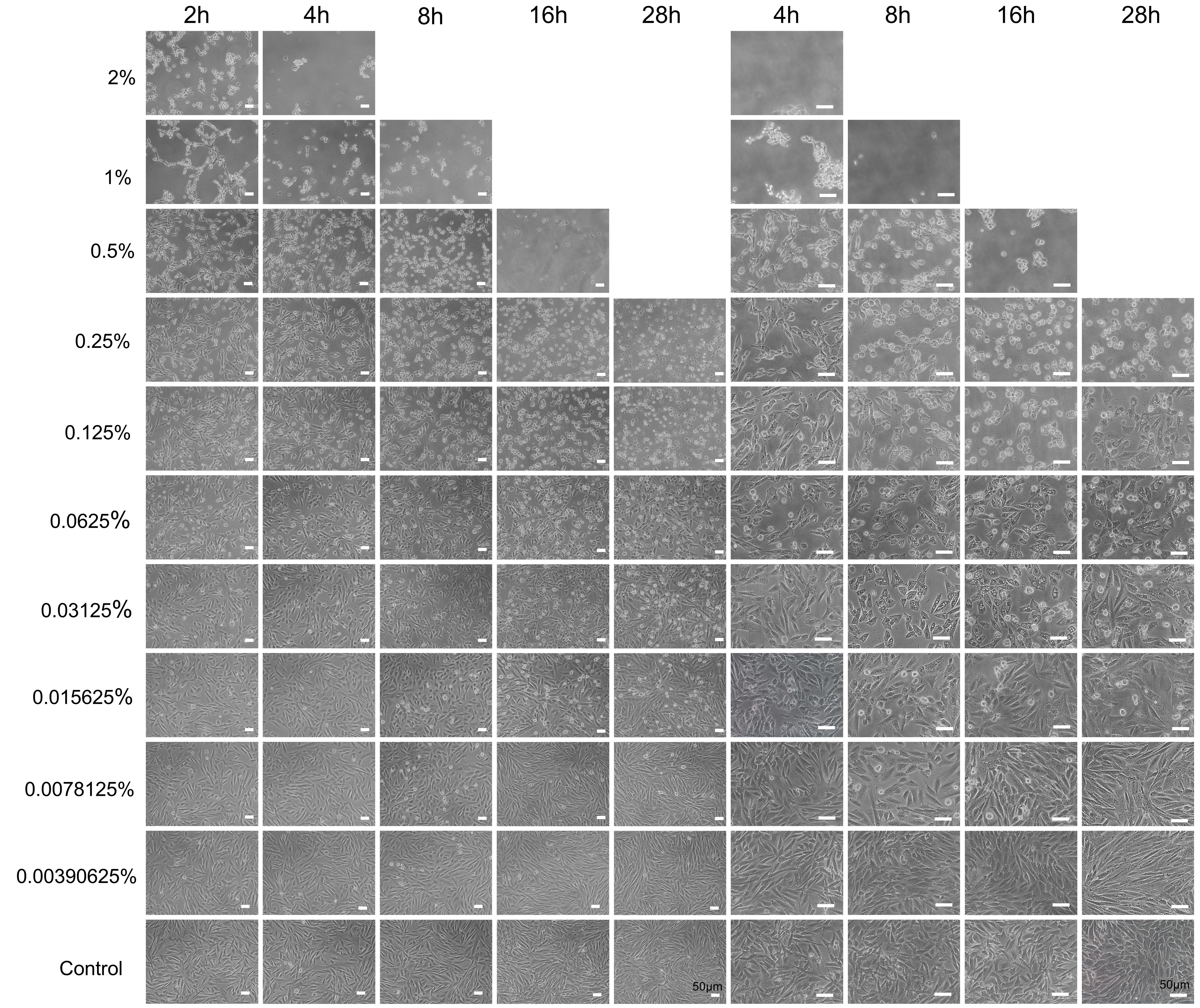

Supplement: FIGURE S1 — Morphological abnormality and growth status of carteolol-treated HCECs. The concentration and exposure time of carteolol were indicated on the top-left of each image. Images represent n = 3. Scale bar is 50 μm. [file Image_1.TIF]

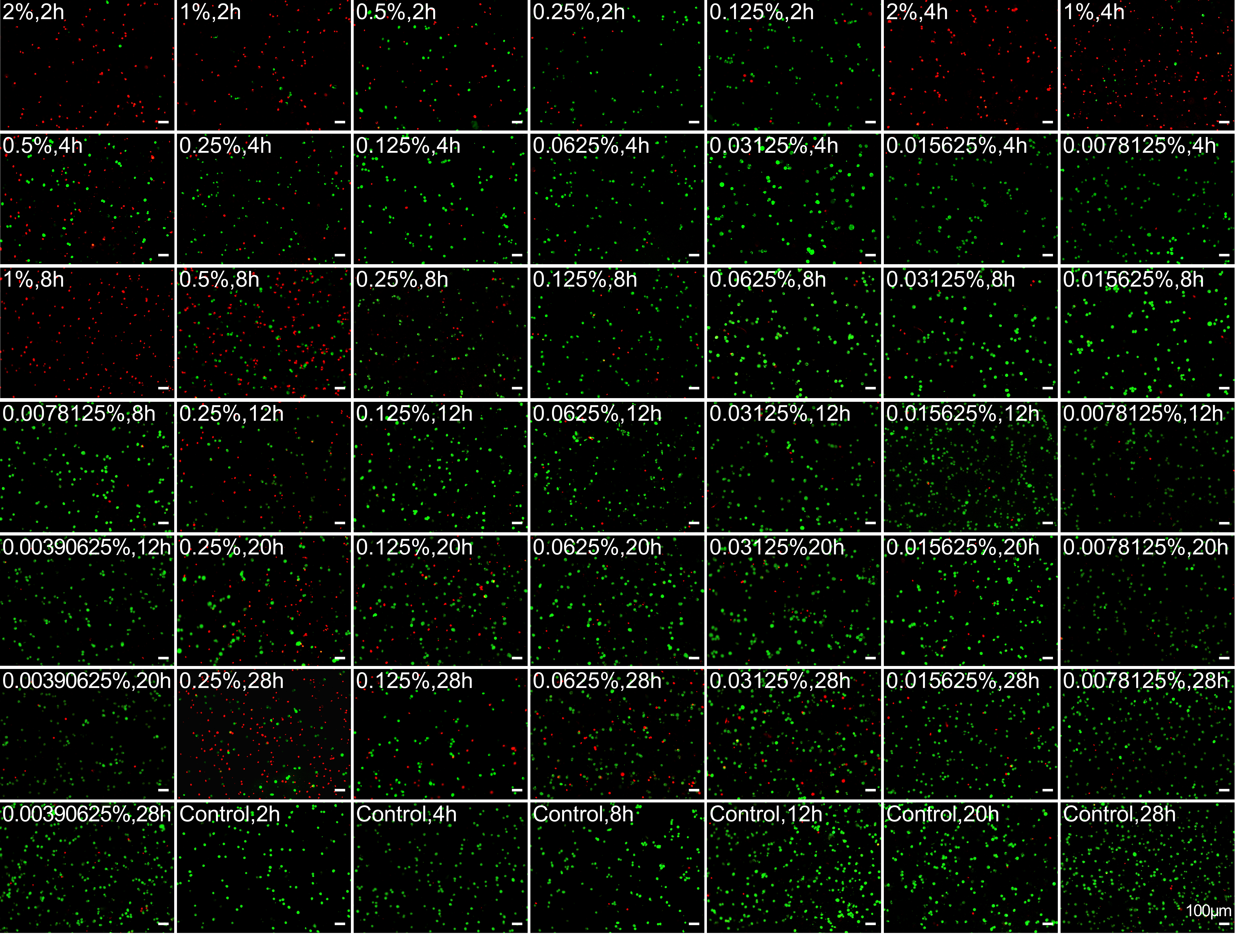

Supplement: FIGURE S2 — AO/EB double-staining images of carteolol-treated HCECs. Scale bar is 100 μm. [file Image_2.TIF]

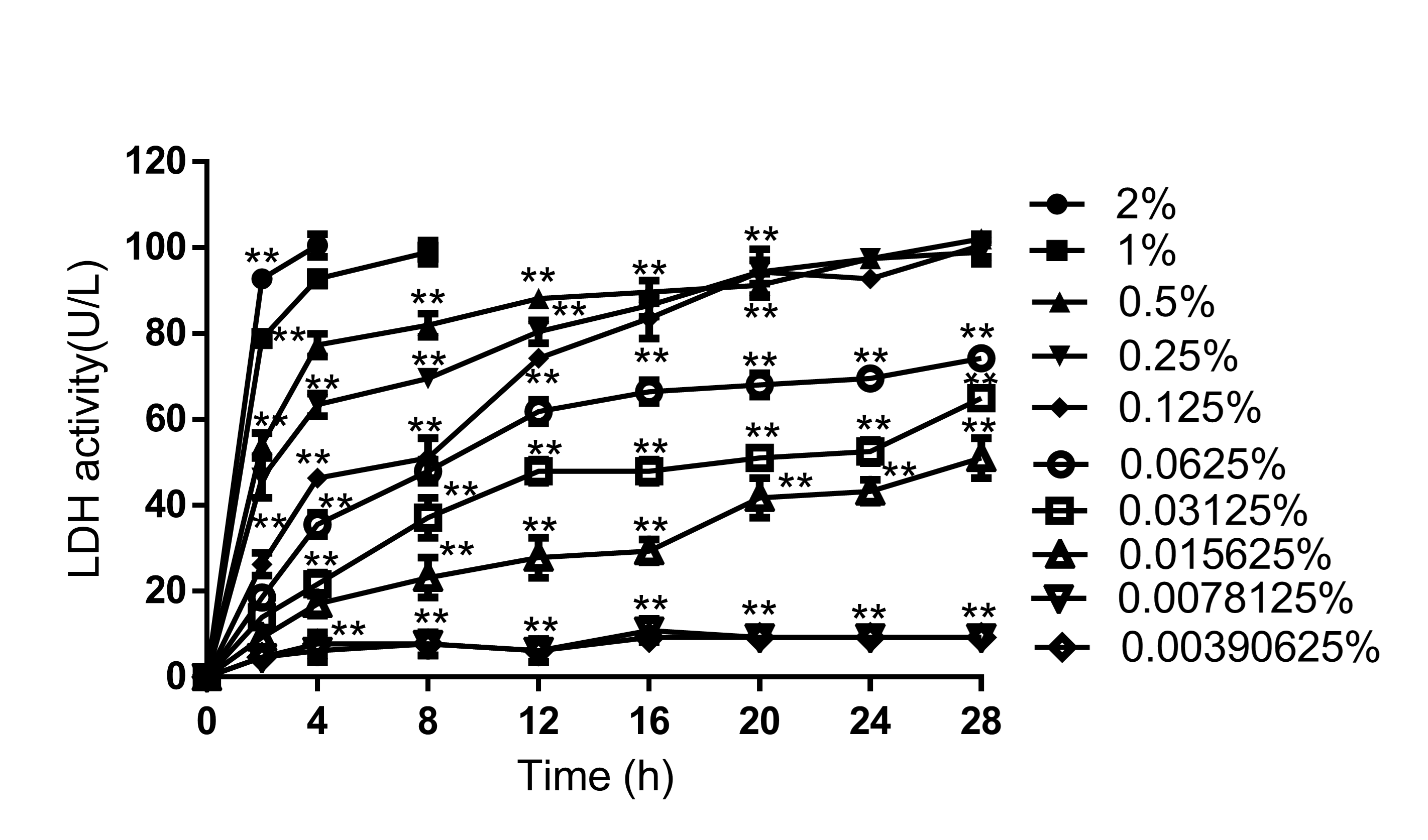

Supplement: FIGURE S3 — LDH double-staining images of carteolol-treated HCECs. [file Image_3.TIF]

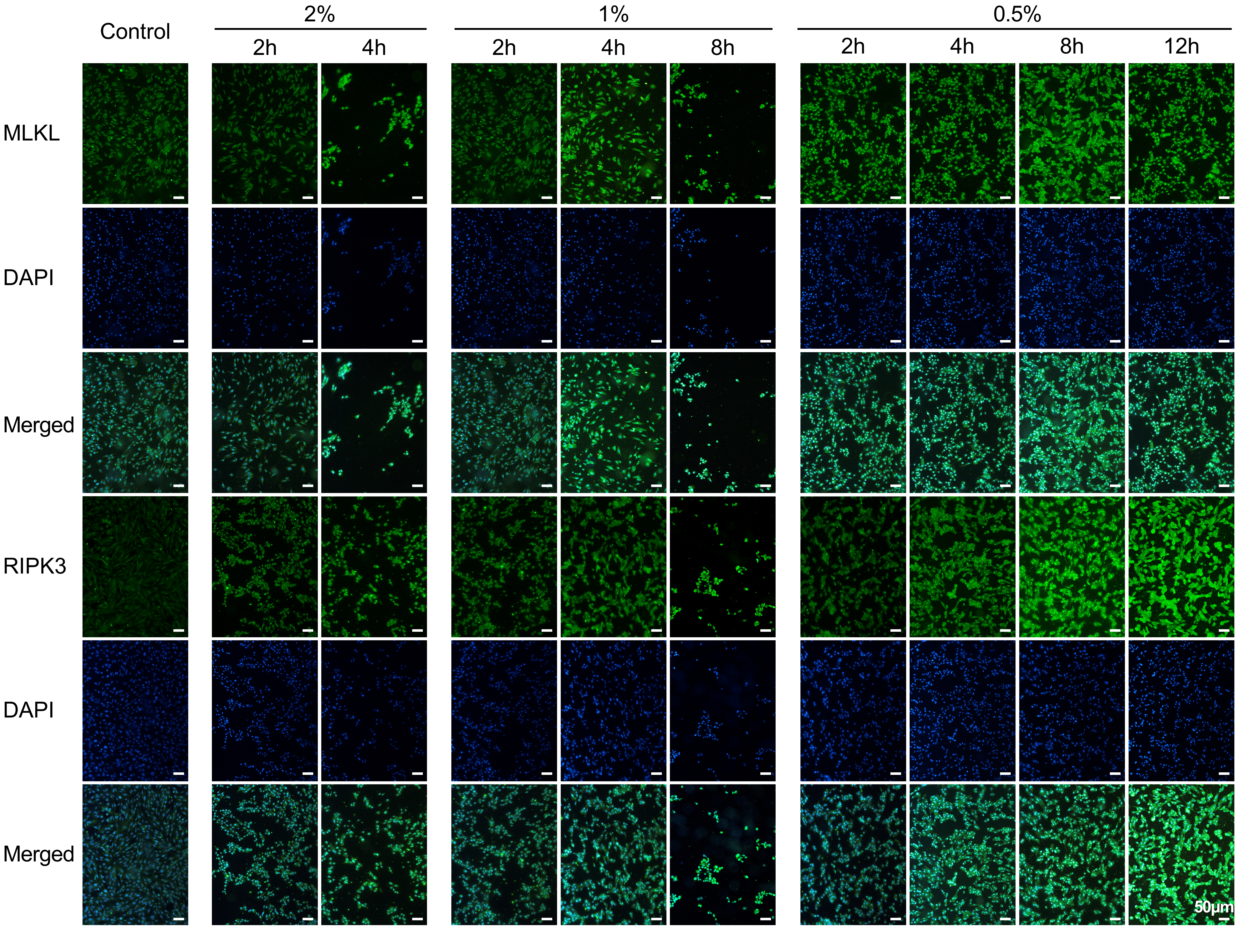

Supplement: FIGURE S4 — Immunochemistry images of high concentration carteolol-treated the expression of RIPK3 and MLKL protein in HCECs. Scale bar is 50 μm. [file Image_4.TIF]

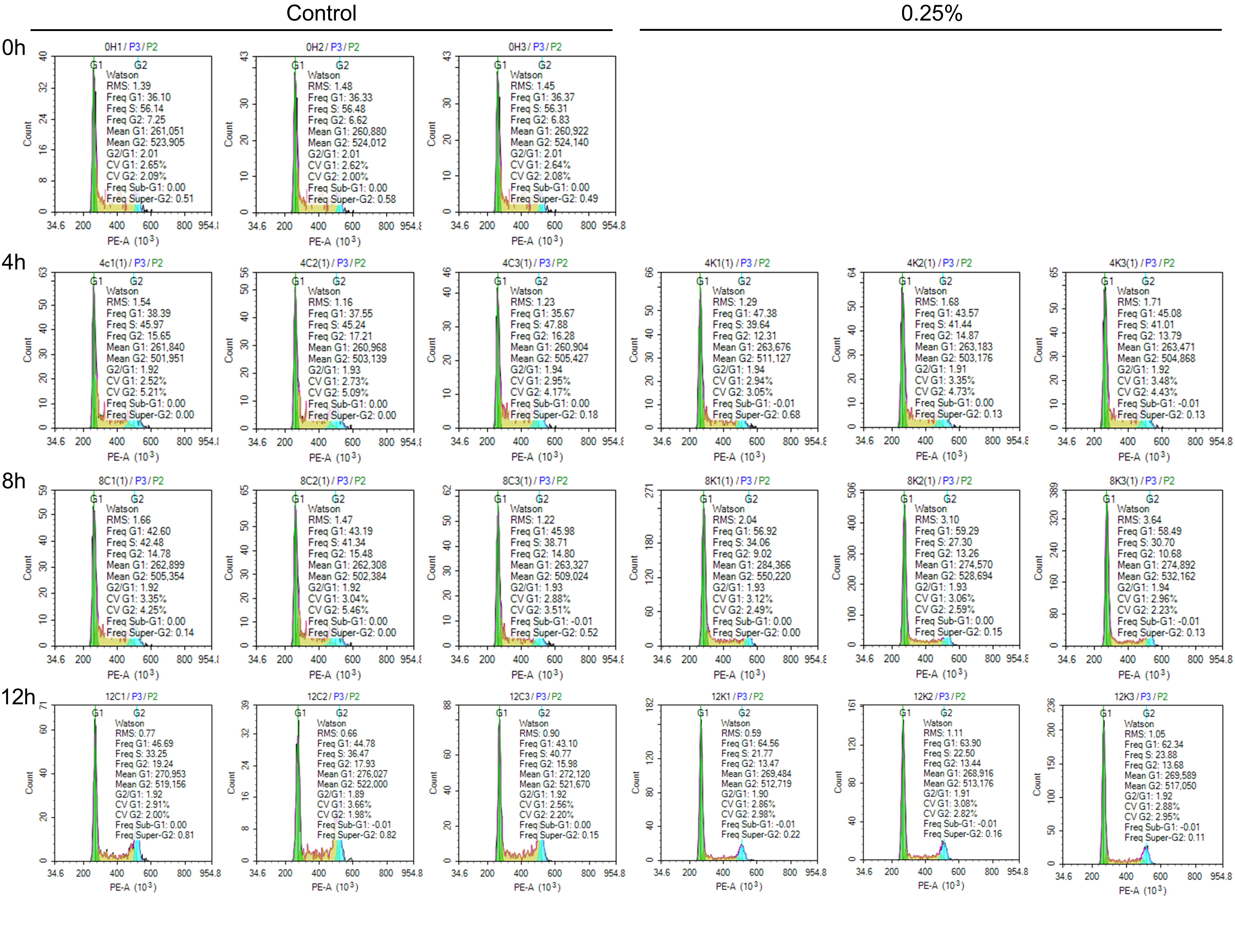

Supplement: FIGURE S5 — PI staining by FCM images of cell cycle parameters in 0.25% carteolol-treated HCECs. [file Image_5.TIF]

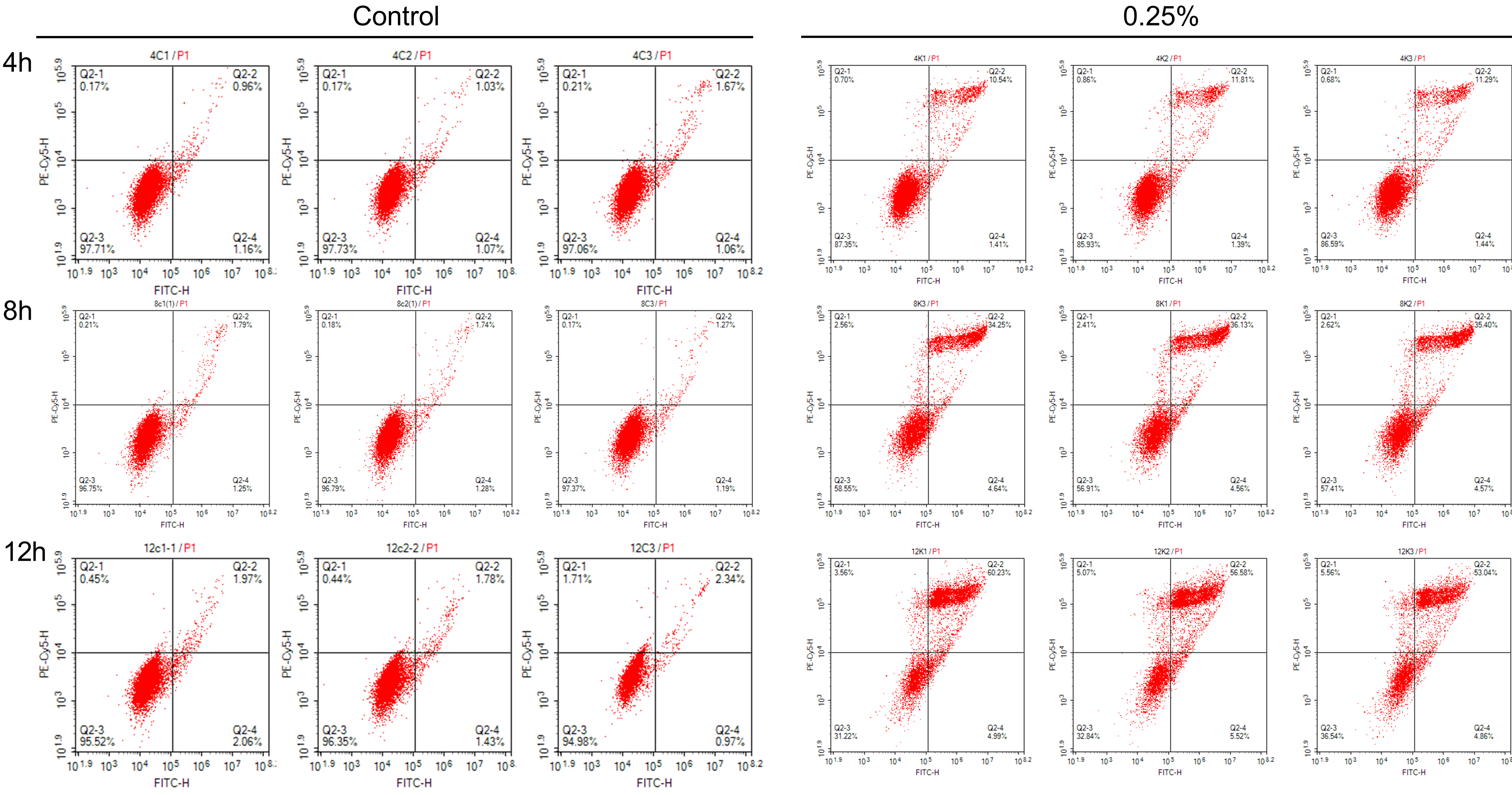

Supplement: FIGURE S6 — Annexin-V/PI staining by FCM images of PS-externalized cells. [file Image_6.TIF]

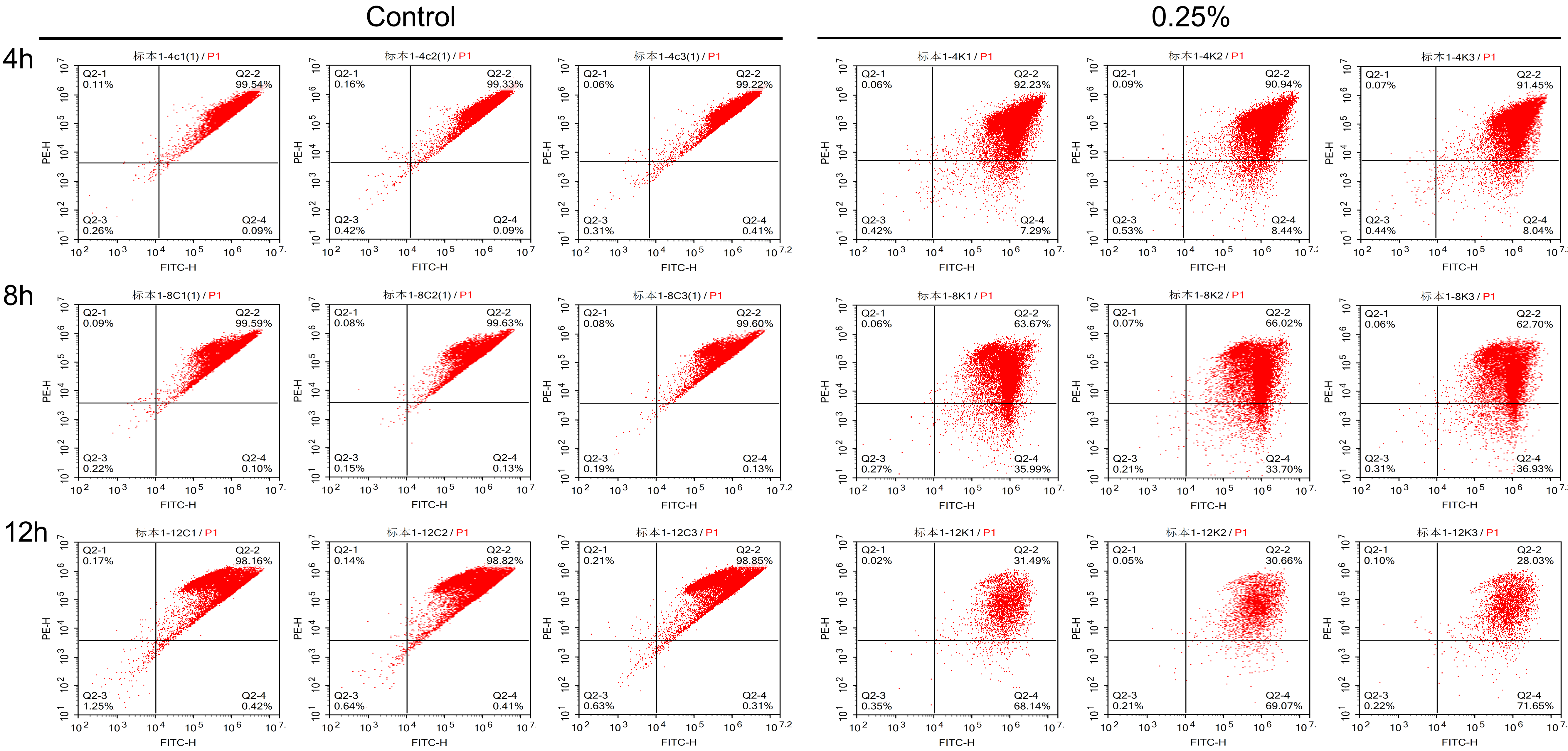

Supplement: FIGURE S7 — JC-1 staining by FCM images of ΔΨm disruption in 0.25% carteolol-treated HCECs. [file Image_7.TIF]
